# Developing a Quality Evaluation System for Color Reproduction of Color 3D Printing Based on MATLAB Multi-Metrics

**DOI:** 10.3390/ma16062424

**Published:** 2023-03-18

**Authors:** Liru Wang, Jiangping Yuan, Qinghua Wu, Guangxue Chen

**Affiliations:** 1 State Key Laboratory of Pulp and Paper Engineering, South China University of Technology, Guangzhou 510640, China; 2 College of Communication and Art Design, University of Shanghai for Science and Technology, Shanghai 200093, China

**Keywords:** color 3D printing, color reproduction, quantitative metric, correlation strategy, quality evaluation system

## Abstract

Color 3D printing has been widely used in many fields such as cultural, medical, industrial, and food. The color reproduction accuracy of 3D printed products in these fields is becoming increasingly demanding, which requires more reproduction methods and practical tools. At present, most color 3D printing devices use one quantitative index, that is, color difference, to directly predict the color reproduction quality. However, this single quantitative index is not optimal for the curved surface of 3D printed color objects. Based on color evaluation principles, in this study, five new quantitative metrics consisting of color gamut comparison index, color SSIM index, color FSIM index, iCID index, and subjective scaling values are proposed for comparison, and the corresponding GUI design and code implementation of new color quality evaluation system are performed by MATLAB. Moreover, the comprehensive color assessment of color 3D printed products is confirmed by utilizing standard image acquisition and microscopic imaging methods that are not limited to printing materials and sampling locations. The operation of this system is validated to provide interactivity, simplicity and high efficiency. As a result, the system can provide new valuable feedback for color separation and output calibration of color 3D printing devices.

## 1. Introduction

In recent years, 3D printing has become a flexible and powerful technology in advanced manufacturing [1]. It has been widely used in many countries for personalized manufacturing and smart fabrication [2,3], which has also led to a huge change in industrial design [4,5], manufacturing methods [6,7], and digital flow [8]. With the diversification of materials and digitization of processes, 3D printing for high fidelity manufacturing of shape and performance is becoming more and more achievable [9,10,11]. One of the key challenges that 3D printing needs to address is color reproduction and its control, which is becoming increasingly popular in multidisciplinary applications [12]. As a result, color 3D printing has become an important branch of the 3D printing industry, which is growing quickly [13].

Currently, color reproduction in 3D printing basically relies on the textured color of the material or adhesive, which is affected by the color conversion algorithm and digital transmission model. For example, Wang et al. explored the color change properties of natural pH-sensitive pigments using an electrolytic method to achieve hue control in 3D printing by adjusting the potential [14]. Meanwhile, Wei et al. presented the response surface methodology to determine the optimal color specification to compensate for the color deviation between the measured color of the sample using PolyJet 3D printing and the target digital color [15]. Subsequently, Lee et al. explored the effects of layer thickness and print orientation on the color stability of 3D printed objects using a spectrophotometric method [16]. Obviously, the current research literature on color 3D printing focuses on offline color reproduction quality evaluation and optimization, and lacks practical online inspection and evaluation tools.

Parallelly, color gamut and chromaticity attributes have emerged as color reproduction quality evaluation indexes in the study of color reproduction influence factors for color 3D printing [17,18]. This is a good inspiration for the current 3D printing community, which relies heavily on a single-color difference as an indicator of color reproduction quality. Furthermore, some preliminary studies have also confirmed that chromatic metrics do not precisely match human vision in terms of color representation for color 3D printed objects, and more evaluation metrics are needed [19,20]. For this reason, image-based metrics have been gradually developed for color 3D printing, such as structural similarity, feature similarity, and image difference, which are full-reference image quality comparison metrics [21,22,23]. These image-based metrics are implemented in statistics, from grayscale map comparison to color map comparison.

In addition, studies on the quality evaluation of the color reproduction of 3D printed products with different substrates have been discussed as part of the objective metric described above. Previously, Liu et al. developed an initial 3D color reproduction system based on 3D scanning and 3D printing based on a polynomial regression color management approach in order to improve its color difference [24]. In fact, the current analysis of color 3D printing color accuracy reproduction challenges also indirectly illustrates the dilemma of the lack of color accuracy measurement methods in existing standard organizations and provides an extended framework for their color reproduction evaluation accuracy improvement that can be evaluated online [25]. At the same time, the color reproduction evaluation of color 3D printing is not embedded in the printing system and there are few available online analysis tools that include these objective metrics. Therefore, the development of a color reproduction quality evaluation system that can automatically analyze data is particularly critical for color 3D printing.

In this paper, a quality evaluation system for the color reproduction of color 3D prints is designed and implemented by MATLAB multi-metrics assigned in different modules. These metrics are composed of the color difference index, color gamut comparison index, color SSIM index, color FSIM index, iCID index, and subjective scaling values, which are also analyzed with different indicators and statistical methods to provide more flexible evaluation options. Section 2 illustrates basic correlation and algorithmic framework of color quality evaluation metrics for color 3D printing. Section 3 proposes the guided interface design and key codes for this evaluation system with specific details. Section 4 tests the effectiveness of the current developed system and discusses its optimization ideas for color quality evaluation.

## 2. Quality Evaluation Metrics for Color Reproduction of Color 3D Prints

### 2.1. Correlation of Color Quality Evaluation Metrics

The number of color quality evaluation indicators has a great impact on the accuracy and efficiency of color reproduction quality evaluation of color 3D printed parts. Color difference is the most commonly used quantitative metric, but does not evaluate arbitrary colored areas of color 3D printed parts very well. As a result, this study proposes three color image-based metrics and a subjective scaling metric for correlation analysis, which can fully reflect the color reproduction characteristics of color 3D printed samples. Three color image-based metrics are the color SSIM value, color FSIM value, and iCID value based on standard imaging conditions and precise devices.

The color difference value is usually calculated using two color difference formulas, CIEDE76 and CIEDE00, with the former being concise and the latter being highly accurate. The image-based metrics count all colors at a certain viewpoint, allowing for a comparison of sample points in different curvature regions. Existing image-based metrics are converted to grayscale maps for statistical analysis, but the image-based metrics used in this study are compiled directly on color maps to evaluate the color differences. The features and weights used in the statistics of different color image-based metrics will be different, and thus three typical metrics are selected for testing and analysis in this study. As the acquisition of the standard image required for color-image-based metrics is more complicated than the acquisition of the sample point color values, this system performs a numerical correlation analysis between the color difference and color image-based metrics on the printed sample data. The surface coloring efficiency (SCE) metric was also based on the physical measurement of the sampling surfaces on the color 3D prints in order to provide new objective assessment. Moreover, the subjective scaling metric is a standard observer’s perceived evaluation of the surface color of color 3D printed parts, and is the basis for the development of all of the current objective evaluation methods.

Overall, this evaluation system contains most key metrics currently relevant to the color quality of color 3D prints, from objective indicators to subjective indicators.

### 2.2. Algorithmic Framework of Color Quality Evaluation Metrics

Based on the multiple metrics required for the color quality evaluation described above, the solution process was decomposed step-by-step and compiled into specific functional modules for color 3D printing of different materials using the 2016a version MATLAB software.

(1) Color difference algorithm

There are many formulas that can be used for point-by-point color difference analysis, but the classical color difference formulas are CIEDE1976 and CIEDE2000 [23].

(1) $${\Delta E}_{76}^{*}\left(O,I\right)=\sqrt{{\left(L_{o}^{*}-L_{i}^{*}\right)}^{2}+{\left(a_{o}^{*}-a_{i}^{*}\right)}^{2}+{\left(b_{o}^{*}-b_{i}^{*}\right)}^{2}}$$

(2) $${\Delta E}_{00}^{*}\left(O,I\right)=\sqrt{{\left(\frac{L_{o}^{*}-L_{i}^{*}}{K_{L}*S_{L}}\right)}^{2}+{\left(\frac{C_{o}^{*}-C_{i}^{*}}{K_{C}*S_{C}}\right)}^{2}+{\left(\frac{H_{o}^{*}-H_{i}^{*}}{K_{H}*S_{H}}\right)}^{2}+R_{T}\left(\frac{C_{o}^{*}-C_{i}^{*}}{K_{C}*S_{C}}\right)*\left(\frac{H_{o}^{*}-H_{i}^{*}}{K_{H}*S_{H}}\right)}$$

where ${\Delta E}_{76}^{*}\left(O,I\right)$ in Equation (1) is the color difference of all color samples of two color 3D reference plates calculated based on the CIEDE 1976 color difference formula, and similarly, ${\Delta E}_{00}^{*}\left(O,I\right)$ in Equation (2) is the color difference of all color of the samples of two color 3D reference plates calculated based on the CIEDE 2000 color difference formula. The L*, a*, b* are the chromaticity values of the test sample.

**Figure 1:**
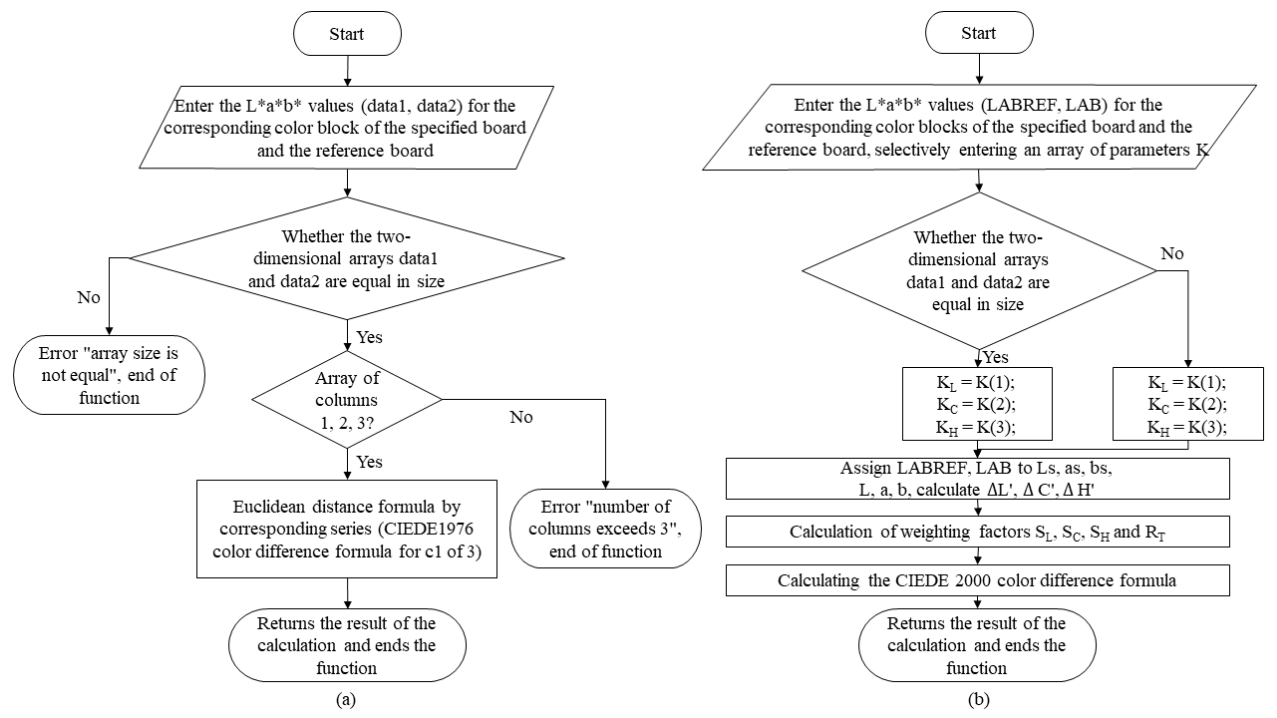
Compilation framework for color difference algorithms: (**a**) CIEDE1976 and (**b**) CIEDE2000.

In order to provide multiple evaluations of the color differences of color samples on the surface of color 3D printed parts, all of them are compiled into the quality evaluation system in this study. Hence, Figure 1 shows the specific compilation flow corresponding to the two algorithms.

(2) FSIM algorithm

**Figure 2:**
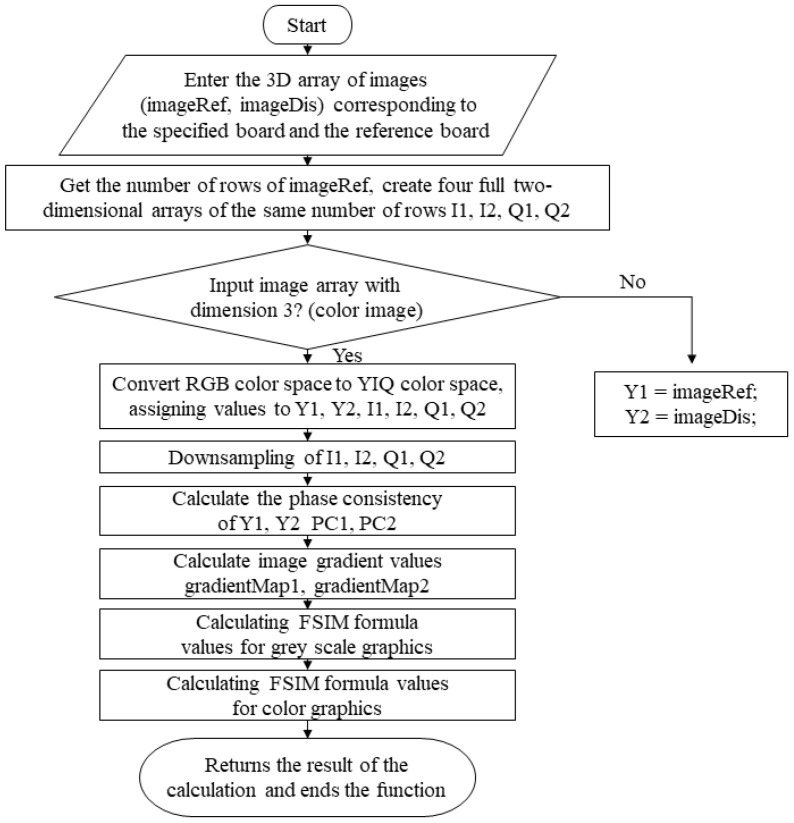
Compilation framework for the grayscale FSIM algorithm.

The FSIM algorithm is a full reference class algorithm commonly used for the objective comparison of the image quality and is used to quantify color differences in feature areas. This study utilizes this metric to quantify the color quality of the main feature region of a standard acquired image under a certain viewpoint of a color 3D printed part, and its corresponding compilation framework is shown in Figure 2. This FSIM algorithm is compiled in the form of a MATLAB function that is oriented to grayscale maps for difference calculation. In the function module of this system, the color channels will be separated for the test images and then quantified by FSIM statistics, which is found in the main function.

(3) SSIM algorithm

**Figure 3:**
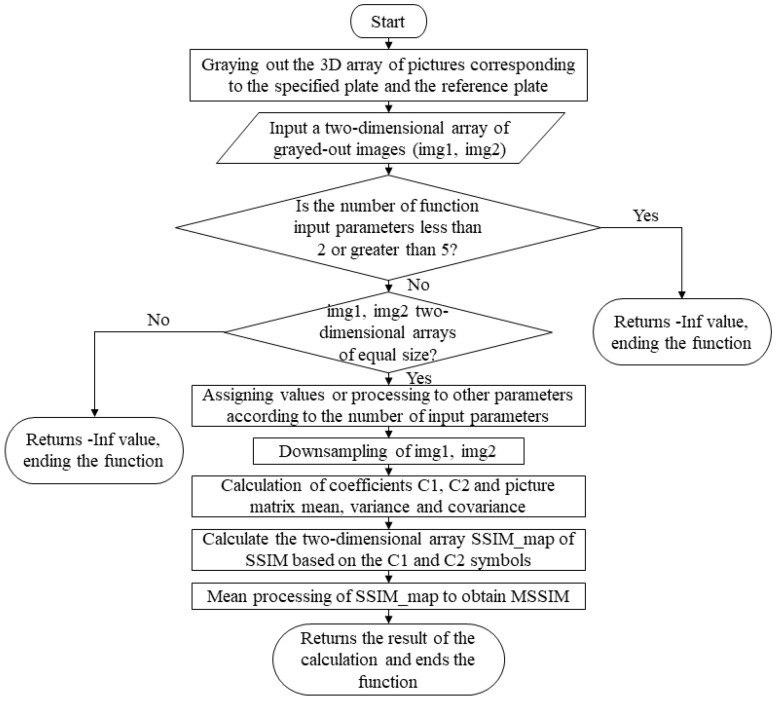
Compilation framework for the grayscale SSIM algorithm.

The SSIM algorithm serves a similar purpose as the FSIM algorithm, but its statistics focus on image structure differences, which are important for color 3D feature comparisons. Figure 3 shows its compilation flow, but its color SSIM value for color image application needs to provide the calling code in the corresponding module of this system.

(4) iCID algorithm

**Figure 4:**
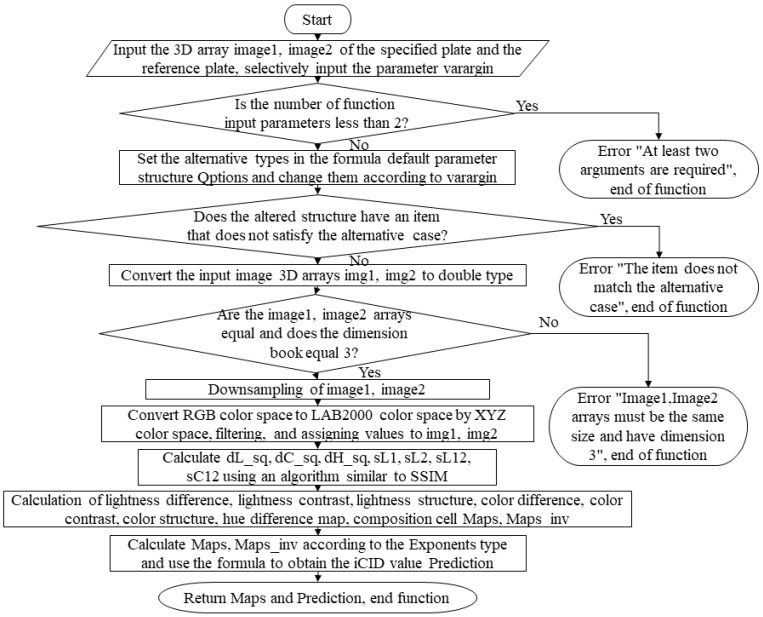
Compilation framework for the iCID algorithm.

The iCID algorithm is a full reference metric that is statistically analyzed by calculating the perception of color differences. Figure 4 shows its compilation framework and numerical statistics. This current algorithm is also called a functional function in the MATLAB system.

(5) SCE algorithm

**Figure 5:**
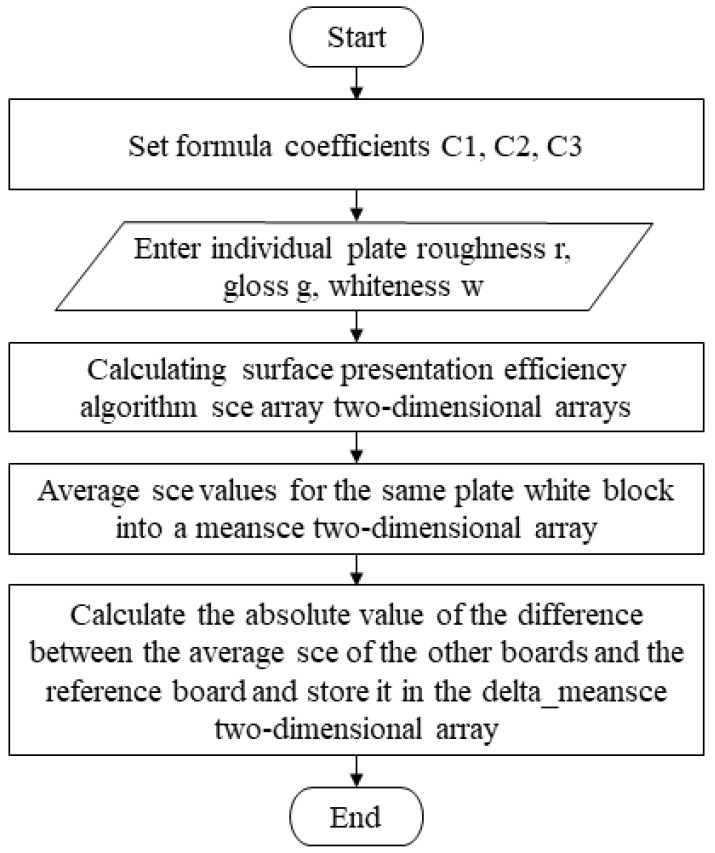
Compilation framework for the proposed SCE algorithm.

The SCE algorithm is an algorithm used to quantify the surface coloring efficiency of color 3D printed parts, mainly considering whiteness, roughness, and transparency. At the same time, the weights of the corresponding surface properties can be set flexibly in the system to match different printing materials, as shown in Figure 5. Thus, it is compiled into the SCE_3d function, which is known as the main program.

(6) Color gamut comparison algorithm

**Figure 6:**
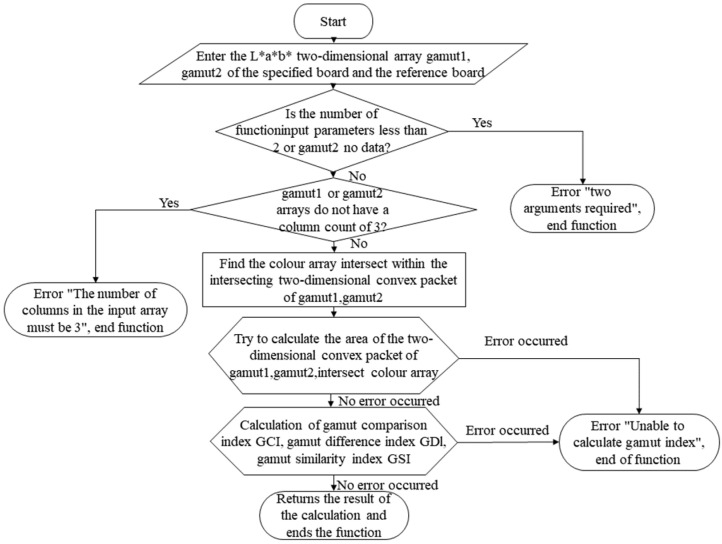
Compilation framework for color gamut comparison algorithms.

The ICC recommends one formula for color gamut comparison algorithm, but this study provides three formulas in conjunction with the team’s previous research [25]. Figure 6 shows its corresponding numerical statistics and compilation framework. In addition, the Venn function is used to make a life-like visual demonstration of the values calculated by the above formula, which can be used in the main program.

(7) Objective correlation algorithm

**Figure 7:**
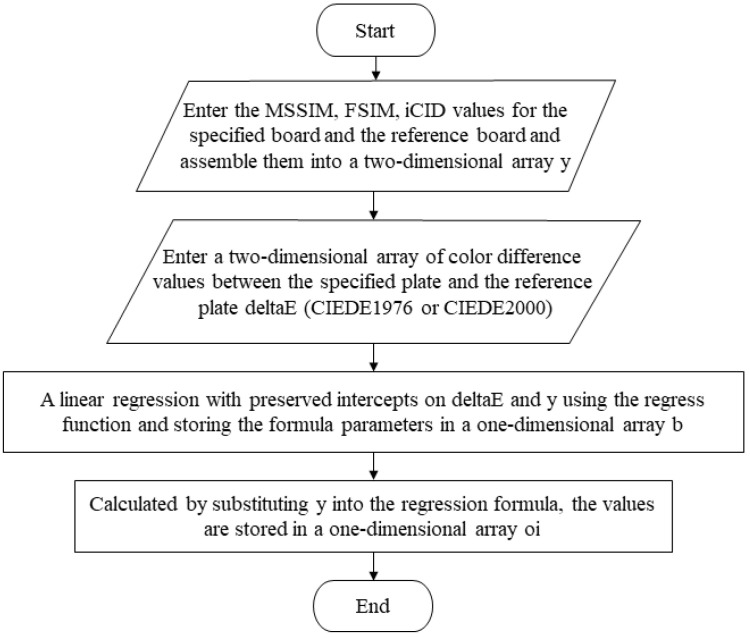
Compilation framework for the objective correlation algorithm.

The integrated evaluation of multiple metrics can achieve a specific balance between efficiency and accuracy. The correlation properties between the current objective five metrics are in need of validation, and alternative fitting metrics that can replace the color difference metric are identified. So, Figure 7 demonstrates the corresponding compilation framework and provides an optimization reference for setting the image-based metric linear fit parameters.

(8) Subjective scaling algorithm

**Figure 8:**
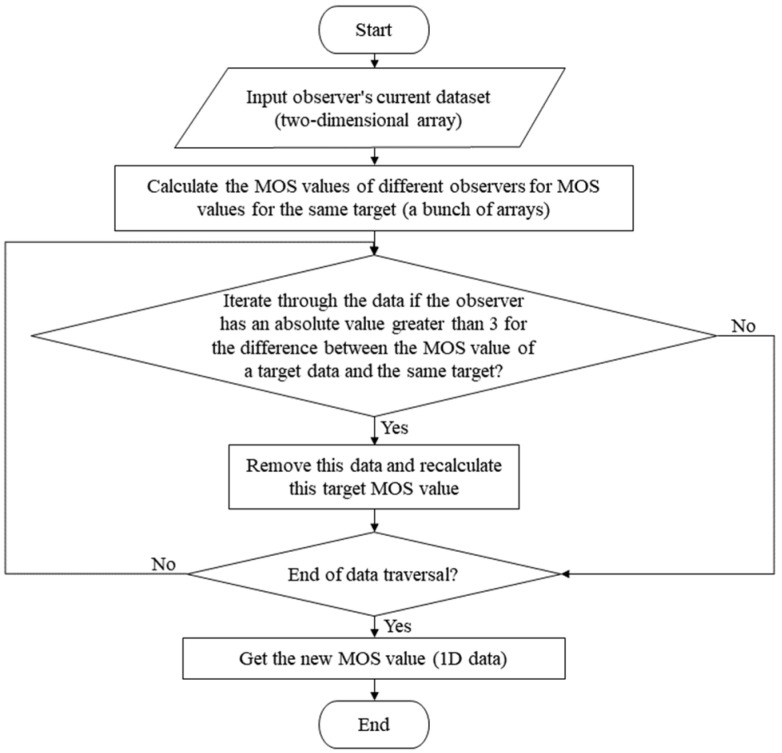
Compilation framework for the subjective scaling algorithm.

Importantly, the subjective scaling algorithm is used for the quantification of color perception differences on physical surfaces and is commonly characterized by the mean opinion score (MOS). Figure 8 illustrates the compiled framework of the current study for the analysis of visual perceptual differences on the surface of colored 3D printed parts.

The current section shows, in detail, the compilation framework of the color quality evaluation algorithms and gives ideas for optimization in color image applications. Some of them are commonly used algorithms and some are innovative metrics of this research team [22,23]; all of them can be compiled into MATLAB functions for different function module calls.

## 3. Developing a Color Quality Evaluation System for Color 3D Printing

**Figure 9:**
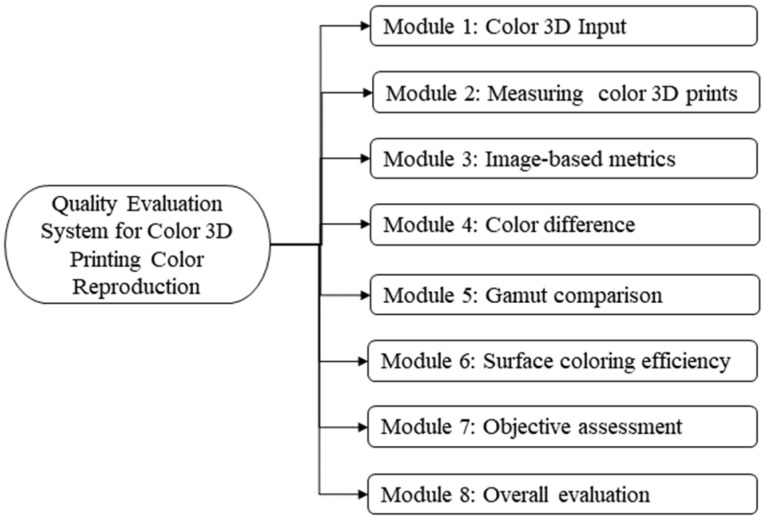
The basic architecture of the proposed system.

Based on above-mentioned metrics and their compilation framework, this section specifically elaborates on the development of a quality evaluation system for color reproduction of color 3D printing. This system will be divided into eight functional modules, each corresponding to one or more of the above metric calculations, and its structural framework is shown in Figure 9. Module 1 and Module 2 are used as data input ports, separately, and the other modules are based on their data for the corresponding metric analysis. Generally, the development of each functional module includes the interface design and the corresponding implementation code.

### 3.1. Interface Design and Demonstration of Module 1

**Figure 10:**
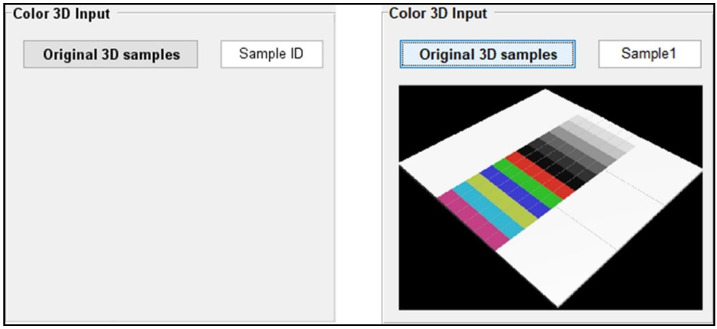
Initial interface and sample demonstration of Module 1.

There are essential structural differences between color 3D printed benchmarks and graphic printed benchmarks, which is the core value of this system being developed for 3D printing community. This module is a relatively simple input function module that provides the user with the flexibility to select test reference samples. Figure 10 shows the initial interface of Module 1 and its Sample1 benchmark file import demonstration. Table 1 also gives details of the types, tags, and roles of the controls used in its interface.

### 3.2. Interface Design and Demonstration of Module 2

**Figure 11:**
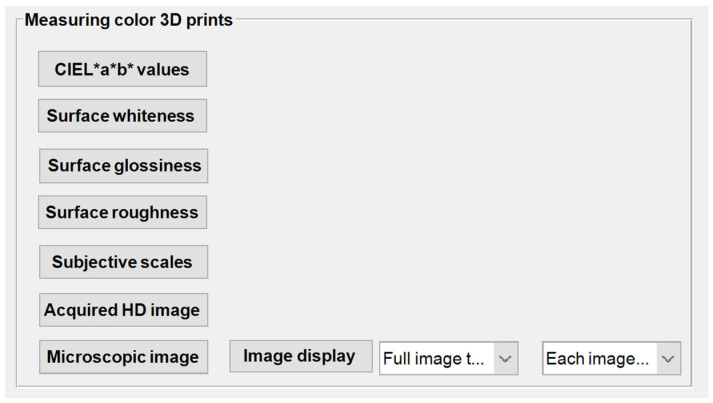
Initial interface of Module 2.

After the benchmark test model is selected in Module 1, the following raw data measurements of the color 3D printed samples can be imported into Module 2, item by item, for preliminary analysis and visualization. This module provides five data sets and two image sets for import to provide the renaming, data visualization, and classification storage of each type of test data, as shown in Figure 11. In addition, Table 2 also gives details about the types, tags, and roles of the controls used in its interface.

**Figure 12:**
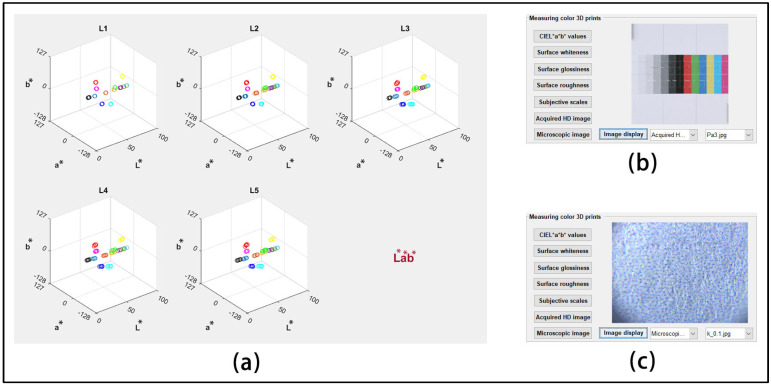
Demonstration of numerical and image import: (**a**) CIEL*a*b* values of five areas; (**b**) high resolution acquired images of Sample 3; (**c**) microscopic imaging of magenta block.

After any category of data is imported, a new window pops up showing five subplots, each containing the measured values for all of the color patches on the benchmark test model or their average values. Figure 12a shows the CIEL*a*b* test data, each subplot corresponds to a color 3D printed test sample, while Figure 12b,c shows a detailed example of the import of two types of image sets. This functional module provides an image display of the macroscopic features and microscopic features to analyze both the state of its coloring material accumulation and the differences in its overall surface light radiation properties. This is another innovative point of the current quality evaluation system.

### 3.3. Interface Design and Demonstration of Module 3

**Figure 13:**
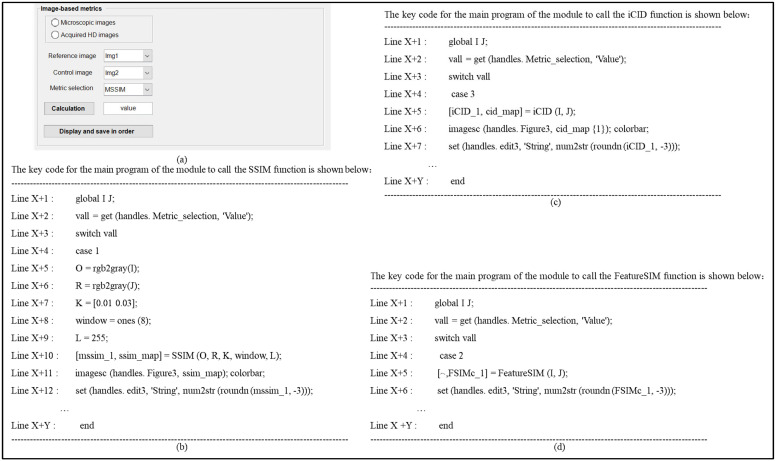
Interface design and demonstration of its calling codes in Module 3: (**a**) initial Interface; (**b**) color SSIM metric; (**c**) iCID metric; (**d**) color FSIM metric.

After all of the image set inputs and global variable calls are set up in Module 2, then Module 3 can start resolving image-based metrics and generating image quality difference maps. This module provides the above three image-based metric comparison operations for two types of image sets, and satisfies the renaming, data visualization, and classification storage of the solved image-based metrics, whose initial interface and key codes are shown in Figure 13. In addition, Table 3 also gives details about the types, tags, and roles of the controls used in the interface.

### 3.4. Interface Design and Demonstration of Module 4

**Figure 14:**
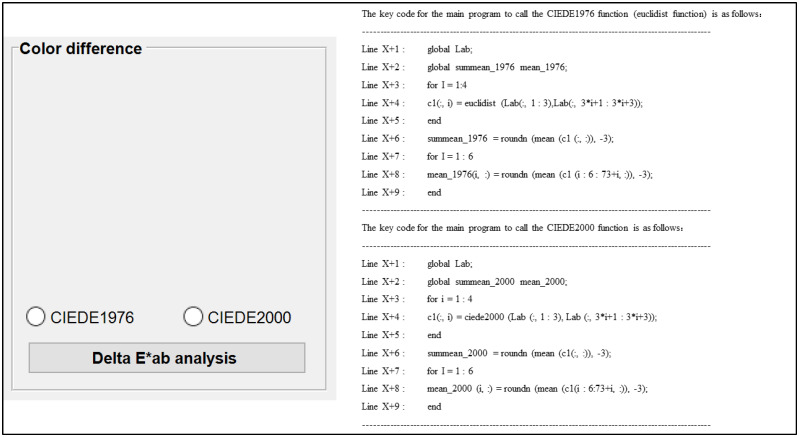
Interface design and demonstration of its calling codes in Module 4.

Starting with Module 4, the function of the modules of this evaluation system is metric analysis and quantitative correlation. The current module is the overall color difference analysis and provides two representative color difference formulas (CIEDE1976 and CIEDE2000) processing codes, shown in Figure 14. Specifically, the control at the bottom of the interface can also provide a specific color difference analysis of the colors of the same height area on each printed benchmark. In addition, Table 4 also gives details of the types, tags and roles of the controls used in its interface.

### 3.5. Interface Design and Demonstration of Module 5

**Figure 15:**
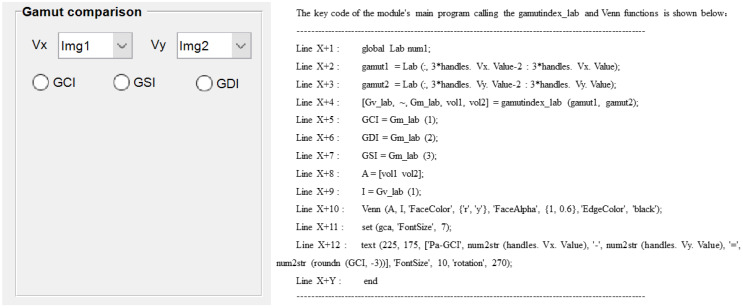
Interface design and demonstration of its calling codes in Module 5.

Module 5 also calls the saved data of Module 2 for color gamut volume calculation, difference comparison and interactive visualization. The comparison order is based on the order of the image set saved in Module 3, and the reference sample name is by default the tested image of the first benchmark. Uniquely, the module provides three color gamut contrast formulas (GCI, GSI, GDI) operating codes for the user to select, shown in Figure 15. In addition, Table 5 also gives details of the types, tags and roles of the controls used in its interface.

### 3.6. Interface Design and Demonstration of Module 6

**Figure 16:**
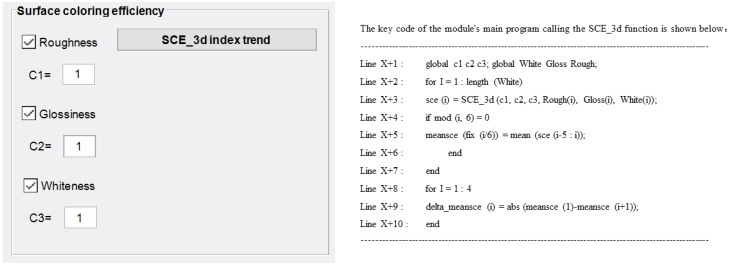
Interface design and demonstration of its calling codes in Module 6.

The SCE metric in this module is not a fixed metric. Module 6 also calls up the whiteness, roughness and gloss data saved in Module 2, combines them with the defined coefficients of the surface coloring efficiency formula, and then calculates the surface coloring efficiency values and visualizes the trends. The module provides the same order of surface coloring efficiency comparison operations as Module 3, and defaults the reference sample name to the first benchmark. Practically, the module provides a mixed selection function for three types of attributes: whiteness, roughness and gloss in Figure 16, and satisfies the flexible setting of weight coefficients for each type of attribute. In addition, Table 6 also gives details of the types, tags and roles of the controls used in its interface.

### 3.7. Interface Design and Demonstration of Module 7

**Figure 17:**
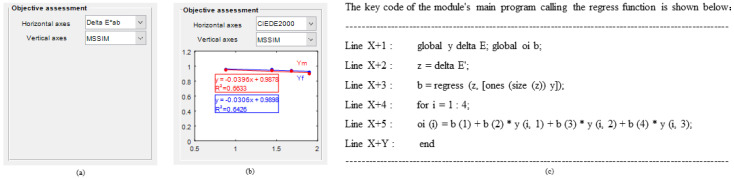
Interface design and demonstration of the calling codes in Module 7: (**a**) initialization; (**b**) terminal; (**c**) code show.

The function of Module 7 is to construct a linear relationship between the overall average of the image-based metrics saved in Module 3 and the overall average color difference saved in Module 4, and to visualize them one by one. Since the order of the overall average color difference operations in Module 4 is the same as the order of the image set saved in Module 3, the two objective measures can be corresponded to each other. The module provides a flexible choice of horizontal and vertical data for linear fitting of objective metrics in the form of the following menus. Interestingly, when the horizontal coordinate data used for subsequent linear fits are the same, the new selected image-based metric data are displayed superimposed in the same coordinate system by codes, shown in Figure 17. In addition, Table 7 also gives details of the types, tags and roles of the controls used in its interface.

### 3.8. Interface Design and Demonstration of Module 8

**Figure 18:**
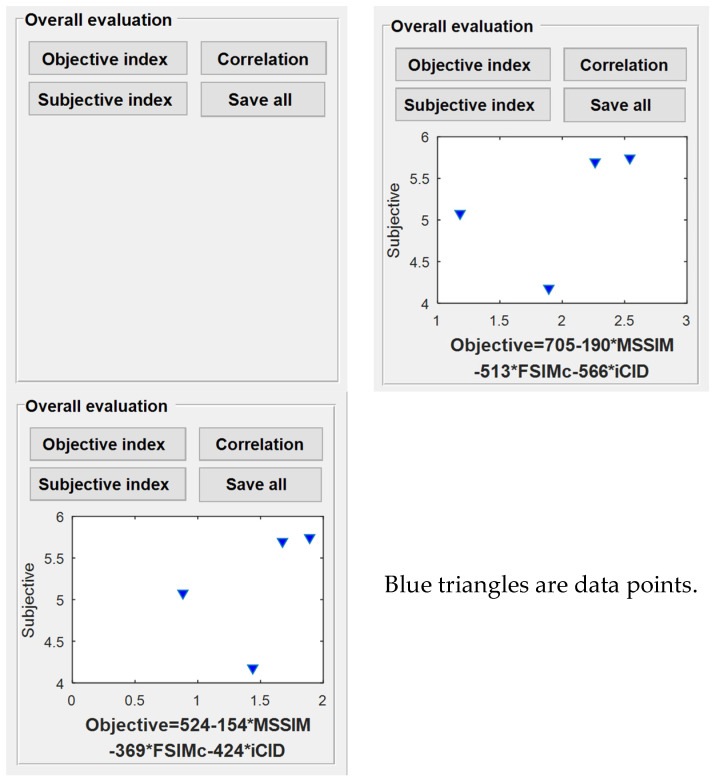
Interface design and its case demonstration in Module 8.

The function of Module 8 is to provide the need for subjective and objective metrics to quantify the correlation visualization and preservation by four control buttons, as shown in Figure 18. The data used in this module for “Subjective index” are the data saved in Module 2, which are used to derive the overall average of the subjective perceptions of each benchmark sample. The data for the “Objective index” are derived from the coefficient values solved by linear regression between the three image-based metrics and the color difference metric in order to construct an integrated image-based metric, and then quantify the trend in the same coordinate system as the requested subjective metric. Based on the color 3D printed samples, Figure 18 also shows their subjective and objective correlation features under the two color difference calculation formulas. In addition, Table 8 also gives details of the types, tags, and roles of the controls used in its interface.

## 4. Results and Discussion

### 4.1. System Operation Test

**Figure 19:**
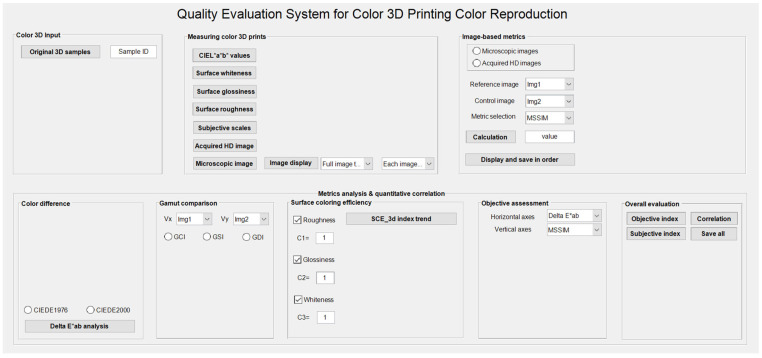
Overall interface design and initialization of the current system.

**Figure 20:**
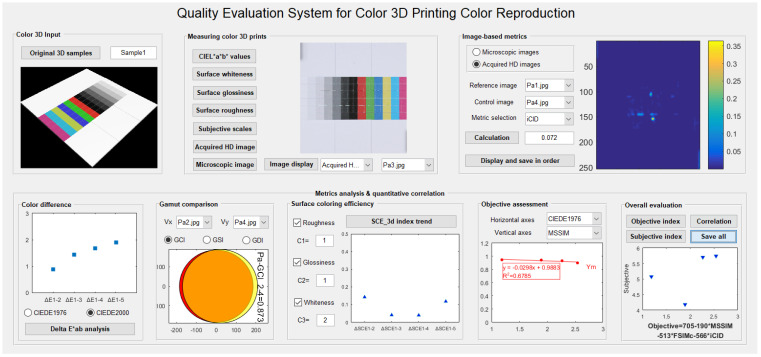
A test case of the current system based on Sample1 and real data in [26].

Figure 19 shows the initial interface details of the developed system, and Figure 20 shows the terminal interface details based on the real data of our published research article using a paper-based color 3D printer [26]. This system provides a very clear view of the output characteristics of each module, and each module is independent and complementary, providing detailed data trends in terms of the color reproduction quality evaluation. Critically, clicking the control button “Save all” stores the current interface state of the entire system, which provides an external reference for color quality evaluation of the color 3D printing sample.

This evaluation system runs smoothly on Win10 PC with more than 5100 lines of total code. Importantly, this system is based on the evaluation behavior of the current color 3D printing color reproduction in industry, and its overall function can also be embedded in the online quality inspection system for color 3D printing. In terms of specific functional implementation, one needs to consider the subjective and objective acquisition features as well as the operational flows, and the other needs excellent architectural design and GUI design. Its architecture is designed to address the smooth transfer of data flow and logic flow between each of the modules, which is a key factor affecting the efficiency of computing and the presentation of results. So, overall interface is designed with eight functional modules that meet the user aesthetics while providing enough detailed feature information to evaluate.

### 4.2. Discussion on the Practicality of the Current System

This system is capable of test benchmark input, measurement data import, analysis result visualization, and interface status output, and its test results based on the test base data of published research papers are the same as those of the manual inference [22,23]. Thus, this subsection will further discuss the derived functions of some modules to expand its application in different color 3D printing devices.

(1) Integrated analysis of image-based metrics using the weighted parameter method

There are many image quality attributes, and the quality evaluation index developed for a particular class of attributes does not accurately apply to all image quality evaluations. This leads to the same problem for the evaluation of high-definition acquired images for specific views of color 3D printed entities. Based on the current color 3D printed benchmark and its measurement data, Table 9 and Table 10 show the linear regression equation and the goodness of fit between the overall color difference and the corresponding image-based metric for the two color difference modes, respectively. It can be found that a change in the mode of color difference calculation leads to a change in the weight coefficients of the constructed linear regression equations, but their numerical expressions are in the same form. In addition, artificial intelligence algorithms may be able to improve the fit when the color difference is associated with fewer image-based metrics for the evaluation analysis.

(2) Optimization of the surface coloring efficiency formula based on microscopic imaging

The setting of the weights of the variable parameters for the evaluation of the surface coloring efficiency in this system can be optimized by quantifying the microscopic imaging features on the surface of the benchmark substrate. Starting from the measurement data of the substrate surface properties, it is possible to circumvent the harsh conditions for accurate color measurement and it also offers the possibility to adjust the coloring properties of the non-printed layer during the printing material distribution. Based on the current evaluation system, the combination of image-based metrics from Module 3 can be quantitatively characterized for the set of microscopic images imported in Module 2 to guide the setting of the weight coefficients for each attribute in the surface coloring efficiency formulation in Module 6. Microscopic image analysis of sampling points on the substrate can qualitatively identify numerical fluctuations in the above surface property measurements, but there is no specific numerical model to guide exactly how to adjust them.

(3) Comprehensive analysis of evaluation results for multiple objective metrics

The current evaluation system provides three types of objective evaluations: image-based metrics for Module 3, chromaticity (color difference and color gamut) for Module 4 and Module 5, and surface coloring efficiency for Module 6, which is quantitatively correlated in Module 7 and Module 8. For color 3D printing applications with uncomplicated color features, the color reproduction quality evaluation of the 3D printed parts can be given quickly and more accurately by using only the color difference metric and color gamut metric, which can be fully satisfied by the current evaluation system. However, when it comes to color 3D printed parts with particularly demanding color reproduction requirements, after acquiring the measurement data required for the calculation of the above three types of objective metrics, it is necessary to reconsider the consistency and preference of the assessment conclusion of each type of objective metric. The goal of the current evaluation system is to be a handy and efficient tool with no sense of autonomous judgment, and it is entirely up to trained color 3D printing engineers to give comprehensive inferences based on the system’s output. This inference can be directly reported to the material assignment system of the color 3D printing device, which in turn can adjust the color sorting parameters to optimize its color reproduction quality. As a result, when encountering disagreement in the evaluation inference of the above three types of objective metrics, this current evaluation system will first refer to chromaticity as the first choice, followed by image-based metrics, and finally to surface coloring efficiency.

## 5. Conclusions

This study comprehensively elaborates on the interface design and code compilation required for the automated evaluation system of color reproduction quality for color 3D printing, and sufficiently discusses the practicality of the current system for the color evaluation of actual 3D printed parts. This evaluation system takes into account the longitudinal height variation of printed color samples, which is fundamentally different from current color reproduction quality evaluation systems in the field of graphic printing in terms of the evaluation of the geometric features of the printed object. The current system can run efficiently, and the next step is to use an artificial intelligence algorithm for accurate fitting of the multi-material data. The number of colors tested by this system is not many, and the subsequent testing of large color gamut entities are also worth exploring in order to improve its evaluation accuracy for multi-material color 3D printing.

## Figures and Tables

**Table 1:** Types, labels, and roles of controls used in the Module 1 interface.

Type of Controls	Tag of Controls	Role of Controls
pushbutton	show	Importing model views from folders, displaying the images and their names in the corresponding positions
axes	Figure 1	Displaying the imported model view in the coordinate system
edit	edit 1	Displaying the name of the imported model view in the text box

**Table 2:** Types, labels, and roles of the controls used in the Module 2 interface.

Type of Controls	Tag of Controls	Role of Controls
pushbutton	lab_value	Display of the CIEL*a*b* values for the specified benchmark test model
pushbutton	whiteness	Display of the whiteness of the specified benchmark test model
pushbutton	glossiness	Displaying the glossiness of the specified benchmark test model
pushbutton	roughness	Display of the roughness of the specified benchmark test model
pushbutton	Subjective_scales	Display of the subjective perception values of the specified benchmark test model
pushbutton	hd_image	Importing a high-definition acquisition image of the specified benchmark test model
pushbutton	mi_image	Importing a microscopic image of the specified benchmark test model
pushbutton	image_display	Making Figure 11 display the image selected in image_type
popupmenu	image_type	Displaying in the drop-down menu the image type just imported
popupmenu	each_image	Displaying the names of all images under the image type selected by image_type in the drop-down menu
axes	Figure 11	Displaying the selected image in the coordinate system

**Table 3:** Types, labels, and roles of controls used in the Module 3 interface.

Type of Controls	Tag of Controls	Role of Controls
radiobutton	radiobutton1	Selecting the target image set and updating the drop-down menu Reference_image with Control_image
radiobutton	radiobutton8	Selecting other image sets and updating the drop-down menu Reference_image with Control_image
popupmenu	Reference_image	Display all image names of the specified image set in the drop-down menu and specify the reference image
popupmenu	Control_image	Displaying all image names of the specified image set in the drop-down menu and specifying the control image
popupmenu	Metric_selection	Selection of the target image base metric algorithm in the drop-down menu
pushbutton	Calculation	Performing operations according to the options in the preceding drop-down menu
pushbutton	pushbutton12	Calculation of the image basis metric solution for the reference image and other control images in the drop-down menu and saving it in the specified excel file
edit	edit3	Display of the values of the most recent operation of the button Calculation
axes	Figure 13	Displaying the corresponding mapping of the image basis solution in the coordinate system

**Table 4:** Types, labels and roles of controls used in the Module 4 interface.

Type of Controls	Tag of Controls	Role of Controls
radiobutton	CIEDE_1976	Calling the CIEDE1976 color difference algorithm to solve for the average color difference value of the coloring steps and the overall average color difference value
radiobutton	CIEDE_2000	Calling the CIEDE2000 color difference algorithm and solving for the coloring step average color difference value and the overall average color difference value.
pushbutton	Delta_E_analysis	Displaying the overall average color difference value in Figure 14 and the coloring step average color difference value in a new pop-up window
axes	Figure 14	Displaying the corresponding overall average color difference value in the coordinate system

**Table 5:** Types, labels and roles of controls used in the Module 5 interface.

Type of Controls	Tag of Controls	Role of Controls
popupmenu	Vx	Designating as a reference sample
popupmenu	Vy	Designating as a control sample
radiobutton	GCI	Displaying the color gamut interaction mapping map and contrast values based on the GCI algorithm in Figure 15
radiobutton	GSI	Displaying the color gamut interaction mapping map and contrast values based on the GSI algorithm in Figure 15
radiobutton	GDI	Displaying the color gamut interaction mapping map and contrast values based on the GDI algorithm in Figure 15
axes	Figure 15	Display the corresponding color gamut interaction map and values in the coordinate system

**Table 6:** Types, labels and roles of controls used in the Module 6 interface.

Type of Controls	Tag of Controls	Role of Controls
checkbox	checkbox1	Whether the SCE formula includes a weighting factor for roughness
checkbox	checkbox2	Whether the SCE formula contains a weighting factor for glossiness
checkbox	checkbox3	Whether the SCE formula contains a weighting factor for whiteness
edit	edit4	Radio checkbox1 to update the weight coefficients of roughness
edit	edit5	Radio checkbox2 to update the weight coefficients for glossiness
edit	edit6	Radio checkbox3 to update the weight coefficients for whiteness
pushbutton	pushbutton14	Display of the overall average SCE difference in Figure 16 and a new window showing the base white block SCE values
axes	Figure 16	Display the overall average SCE difference within the coordinate system

**Table 7:** Types, labels, and roles of controls used in the Module 7 interface.

Type of Controls	Tag of Controls	Role of Controls
popupmenu	horizontal_axes	The first drop-down menu provides a choice of color difference calculation methods
popupmenu	vertical_axes	The second drop-down menu provides the image basis metric method and displays the linear fit results and their storage in the specified excel
axes	Figure 17	Display multiple sets of linear fit results within the coordinate system

**Table 8:** Types, labels, and roles of controls used in the Module 8 interface.

Type of Controls	Tag of Controls	Role of Controls
pushbutton	pushbutton16	Linear regression with image base metric data according to the color difference selected by the drop-down menu horizontal_axes
pushbutton	pushbutton17	Importing subjective perceptual metric data
pushbutton	pushbutton18	Causing a scatter plot of the subjective and objective data to be displayed in Figure 18, displaying the regression equation under the plot
pushbutton	pushbutton19	Saving the current interface of the system
axes	Figure 18	Display the scatter plot of subjective and objective data in the coordinate system

**Table 9:** Linear regression equation using the CIEDE1976 color difference mode.

Element	Linear Regression Equation	$R^{2}$
MSSIM	Delta E_76_ = −22.761 MSSIM + 23.128	0.678
FSIMc	Delta E_76_ = −28.595 FSIMc + 28.986	0.661
iCID	Delta E_76_ = 21.948 iCID + 0.363	0.641
MSSIM + FSIMc	Delta E_76_ = −80.983 MSSIM + 74.230 FSIMc + 7.116	0.697
MSSIM + iCID	Delta E_76_ = −198.963 MSSIM − 175.109 iCID + 199.734	0.817
FSIMc + iCID	Delta E_76_ = −532.992 FSIMc − 393.479 iCID + 534.338	0.839
MSSIM + FSIMc + iCID	Delta E_76_ = −190.382 MSSIM − 512.662 FSIMc − 566.191 iCID + 704.742	1.000

**Table 10:** Linear regression equation using the CIEDE2000 color difference mode.

Element	Linear Regression Equation	$R^{2}$
MSSIM	Delta E_00_ = −16.768 MSSIM + 17.059	0.663
FSIMc	Delta E_00_ = −20.999 FSIMc + 21.312	0.643
iCID	Delta E_00_ = 16.122 iCID + 0.293	0.623
MSSIM + FSIMc	Delta E_00_ = −72.394 MSSIM + 70.920 FSIMc + 1.762	0.693
MSSIM + iCID	Delta E_00_ = −160.525 MSSIM − 142.865 iCID + 161.147	0.829
FSIMc + iCID	Delta E_00_ = −385.248 FSIMc − 284.150 iCID + 386.250	0.809
MSSIM + FSIMc + iCID	Delta E_00_ = −154.353 MSSIM − 368.765 FSIMc − 424.176 iCID + 524.406	1.000
